# Modeling meningioma in vitro in the omics era

**DOI:** 10.1007/s13577-026-01448-3

**Published:** 2026-09-08

**Authors:** Daisuke Sato, Satoru Miyawaki, Yu Sakai, Yosuke Kitagawa, Hiroki Hongo, Yudai Hirano, Yu Teranishi, Shotaro Ogawa, Takahiro Tsuchiya, So Hirata, Hideaki Ono, Nobuhito Saito

**Affiliations:** Department of Neurosurgery, The University of Tokyo Hospital, 7-3-1 Hongo, Bunkyo-Ku, Tokyo, 113-8655 Japan

**Keywords:** Meningioma, Culture, Cell line, IOMM-Lee, Assay

## Abstract

Meningioma biology has been substantially clarified by recent omics-based studies, which have identified recurrent mutations, copy-number alterations, and distinct molecular subgroups. However, although these approaches have provided a valuable framework, they are inherently limited in their ability to establish direct causal relationships. The mechanistic studies are therefore indispensable for translating these molecular observations into biological understanding. Nevertheless, the mechanistic literature has often evolved in a fragmented manner, with individual pathways and model systems studied in relative isolation from the broader multi-omic landscape. In this review, we synthesize these complementary bodies of work into an integrated framework and outline a clear roadmap for future studies. We first review the historical development of established meningioma cell lines, their current molecular characterization, and the recent emergence of 3D models and organoids. Intrinsic challenges in modeling meningioma in vitro are discussed, including the difficulty of establishing immortalized cell lines from predominantly benign tumors, genetic alterations introduced during immortalization, and drift under culture conditions that differ substantially from those of the parental tumors. Next, insights from functional studies centered on these models are integrated within the molecular framework established by large-scale omics analyses. To avoid fragmentation and overemphasis on isolated findings, prior studies are organized into six categories based on major signaling pathways: Hippo, PI3K/Akt/mTOR, MAPK, Wnt/β-catenin, FOXM1, and Notch. Finally, lessons from other cancer models, including experimental approaches to chromosome-scale genomic disturbances, are considered to provide a more integrated view of meningioma biology and to highlight directions for future research.

## Introduction

Meningioma is the most common primary intracranial tumor, accounting for approximately one-third of all cases [1, 2]. Meningioma encompasses a broad histopathological spectrum of 15 subtypes, about 20% of which are classified as World Health Organization (WHO) grade 2 or higher [3]. Recent technological advancements have profoundly reshaped our understanding of its genomic landscape. Beyond the prevalence of *NF2* mutations, high-throughput sequencing has identified mutually exclusive alterations in non-*NF2* meningiomas, including *TRAF7*, *KLF4*, *AKT1*, *SMO*, *PIK3CA*, and *POLR2A* [4–7]. Aggressive biological behavior is further defined by *TERT* promoter mutations and *CDKN2A/B* homozygous deletions, both of which are now incorporated into the WHO grading system [3, 8, 9]. Also, *BAP1* deficiency [10], *PTEN* alteration [11, 12], H3K27-trimethylation loss [13, 14], dysregulated PRC2 activity [13, 15], and FOXM1 upregulation [13, 16–18] have been linked to aggressive behavior. In parallel, chromosome-scale genomic disturbances are increasingly recognized as central to tumor evolution, and the significance of 22q loss, 1p loss, 1q gain, 10q loss, 14q loss, and 18p/q loss has been repeatedly demonstrated [12, 19–26].

Although these advances have substantially refined our understanding and several rigorous studies have provided insights that extend toward causal biological interpretation [16, 18, 19, 27–35], moving from these observational associations to direct mechanistic or causal inference remains a major challenge. While the *TRAF7/KLF4* co-mutation has been mechanistically linked to tumorigenicity via MAPK signaling activation [36], some of the above non-*NF2* mutations may be passengers or modifiers rather than drivers [37–39]. In parallel, experimental wet studies have yielded important mechanistic insights into selected molecules and pathways, yet these efforts have often been fragmented, addressing relatively localized biological questions. Only a limited number of studies have successfully integrated these dry and wet approaches within a coherent mechanistic framework [18, 40–45].

Also, only a limited number of established models are currently available for meningioma [46]. The diversity of molecular alterations observed in human tumors is insufficiently reproduced in these models [36], the molecular classifications established by recent multi-omic studies are only discussed in limited cases [18, 42], and the chromosome-scale genomic disturbances that appear central to meningioma evolution are not faithfully recapitulated [47, 48].

Furthermore, current treatment options for meningiomas are still largely limited to surgical resection and radiotherapy [49]. Effective therapeutic options for refractory cases remain scarce and a subset of tumors with aggressive behavior follows a devastating clinical course characterized by locally invasive destruction or metastatic spread [50], underscoring the urgent need for the development of effective therapies through rigorous research.

Against this background, an integrative understanding of meningioma biology is essential. Experimental studies have provided important mechanistic insights, but these findings should be interpreted within the molecular framework established by recent multi-omic analyses. In this review, we first summarize the characteristics of established meningioma cell lines and their methods of generation. We discuss their limitations and shortcomings and highlight the emerging potential of three-dimensional (3D) models and organoids. Next, we summarize the mechanistic insights and therapeutic possibilities emerging from these models, placing them in the context of the molecular landscape defined by recent large-scale multi-omic studies. Finally, we introduce model systems from other diseases that address chromosome-scale genomic instability and replication stress, and discuss future directions for research using meningioma cell line models.

### Established meningioma cell lines

**Fig. 1:**
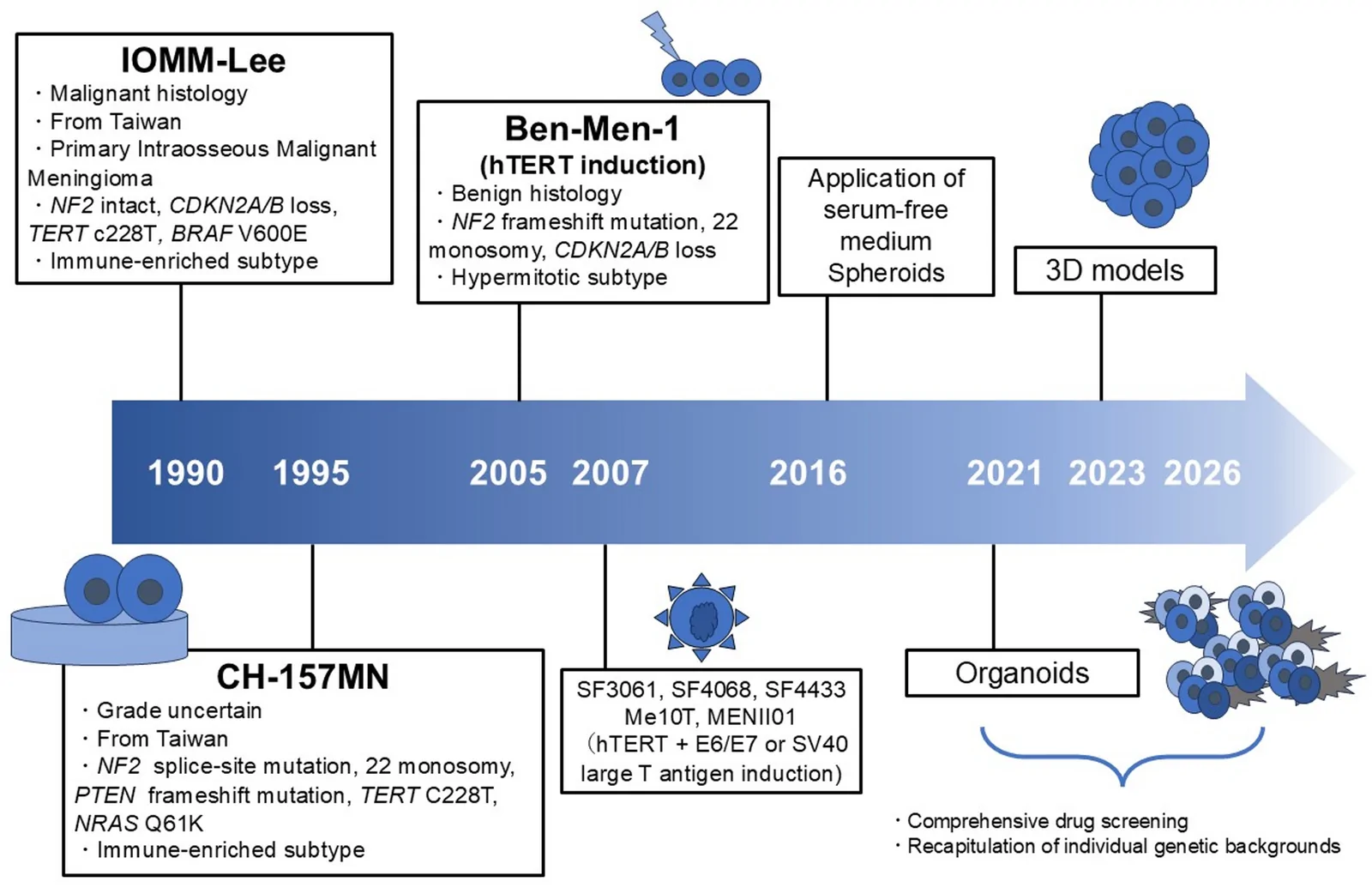
Timeline of in vitro meningioma research. Historical development of in vitro meningioma models, highlighting classical cell lines, later model systems, and representative studies. Early models were limited and largely derived from malignant subtypes, including well-established cell lines such as IOMM-Lee and CH-157MN. The development of genetically engineered models (e.g., Ben-Men-1) was followed by rapid advances in three-dimensional culture systems and organoid models

The first immortalized human cancer cell line was established in 1951 at The Johns Hopkins Hospital [51]. This cell line is now widely known as HeLa. In the field of meningioma research, it was not until the establishment of KT21-MG1 in 1989 and IOMM-Lee in 1990 that the first generation of widely distributable, spontaneously immortalized malignant models became available (Fig. 1). Unlike many aggressive carcinomas, meningiomas generally exhibit slow growth and a high propensity for early senescence in vitro, which has hindered the establishment of stable cell lines.

#### 1980s–1990s: dawn of the meningioma cell lines

This early phase of meningioma cell line research was marked by the establishment of only a few models, mostly derived from malignant subtypes. Importantly, routine testing for mycoplasma contamination and short tandem repeat profiling to exclude cross-contamination had not yet become standard practice [52]. Moreover, immortalization relied on endogenous *TERT* expression [53], as no genetic manipulation–based approaches had yet been introduced.

KT21-MG1 is the oldest cell line we could identify in the literature [54]. This cell line, derived from a malignant meningioma involving the falx, was reported from Japan in 1989. The cell line was described as harboring the pathognomonic monosomy 22. Fresh tumor tissue was xenotransplanted into nude mice (KN-21), where it formed a subcutaneous tumor, and subcultured. Hence, this model was not established entirely through direct in vitro culture.

IOMM-Lee is one of the best-known and most widely used immortalized meningioma cell lines, which was reported in 1990 from Taiwan [48]. This tumor was a primary intraosseous tumor, and the name IOMM is derived from its origin, “IntraOsseous Malignant Meningioma”. The patient presented with a progressive protruding skull tumor and underwent surgical resection followed by postoperative irradiation, after which fresh tumor fragments were obtained. The cell line was cultured in RPMI-1640 supplemented with 20% fetal bovine serum, antibiotics, and L-glutamine. Karyotypic analysis showed an intact chromosome 22, along with numerous structural alterations involving chromosomes 2, 5, 9, 12, 14, 19, and 20. Loss of chromosomes 17 and Y was observed, and an isochromosome 7 was also identified. The cells lacked expression of EMA, cytokeratin, and actin. Later studies further identified *CDKN2A/B* homozygous deletion and *TERT*p C228T mutation, whereas *NF2* remained intact. *BRAF* V600E mutation has been detected [55], which is uncommon in meningioma. Despite its malignant behavior in vivo, epigenetic classification assigns this cell line to the immune-enriched subtype, which is typically *NF2*-altered and benign [18, 42]. Thus, despite its widespread use, this cell line does not recapitulate the most characteristic copy-number alterations (CNAs) or driver mutations commonly observed in meningioma. Moreover, the fact that it originated intraosseously further distances it from the vast majority of meningiomas.

CH-157MN is another well-known immortalized meningioma cell line. It was reportedly established in Taiwan in 1977 and was later described in 1995, though with an undescribed WHO grade [56]. The cells were maintained in a mixture of DMEM and Ham’s F-12 medium supplemented with L-glutamine and were cultured without routine antibiotics. This report excluded Mycoplasma contamination by Hoechst 33,258 staining, an assessment that was not routinely performed in other meningioma cell line research at that time [57]. In contrast to IOMM-Lee, CH-157MN harbors a canonical *NF2* splice-site mutation (c.241-1G>T) and monosomy 22, together with additional chromosome-scale numerical abnormalities, including 1q gain and loss of chromosome 18. Furthermore, this cell line harbors a *PTEN* truncating mutation (p.L57Wfs*42) and a *TERT* promoter C228T mutation, which may have contributed to its immortalization [55]. However, this tumor also harbored an *NRAS* Q61K missense mutation, which is unusual in meningioma. Also, CH-157MN is classified as the immune-enriched subtype by methylation profiling, despite its malignant nature [18, 42]. Notably, the original report described CH-157MN as being derived from a 41-year-old female patient [56], whereas subsequent publications reported donor ages of 59 [58] and 55 years [59], respectively. Because the available literature does not allow this discrepancy to be resolved, the precise donor age remains uncertain. Researchers should therefore be aware of this inconsistency when interpreting studies using this cell line.

#### 2000s: genetically immortalized benign models

This phase of meningioma cell line research, particularly in the late 2000s, was characterized by attempts to immortalize benign meningioma subtypes through genetic manipulation.

Several studies based on short-term primary cultures of benign histologies were published during this period [60], including HBL-52 [61], which was derived from a WHO grade 1 transitional meningioma. However, neither its passage history nor its immortalization method was clearly described. Later studies identified *TRAF7* mutations as characteristic genetic alterations in this cell line [47].

HKBMM is a cell line reported from Japan in 2004 [62]. This was established from a malignant meningioma involving the frontal lobe. Gain of chromosome 21p was described in the original article, although this chromosome is an acrocentric one. The cells were cultured in Ham’s F-12 supplemented with 10% fetal bovine serum and antibiotics, and were reported to proliferate for more than 5 years. Subsequent characterization revealed a *TP53* P177L mutation in this cell line [63], although *TP53* alterations are relatively uncommon in meningioma [64, 65].

The late 2000s saw increasing use of hTERT in combination with viral oncogenes for immortalization. Ben-Men-1, reported in 2005, is the best-known benign meningioma cell line with relatively well-characterized genetic features [66]. Retroviral transduction of hTERT was performed, and the coexistence of an *NF2* frameshift mutation (p.D213fs) and monosomy 22 made this cell line a useful model for studying benign meningiomas. However, this cell line was later found to harbor a homozygous deletion of *CDKN2A/B*, which would classify it as WHO grade 3 under current diagnostic criteria [67].

SF3061, SF4068, SF4433, Me10T, and MENII01 are known to have been immortalized by retroviral transduction of hTERT together with E6/E7 [68, 69]. Whereas hTERT maintains telomere length and thereby bypasses replicative senescence, E6 promotes degradation of p53, and E7 inhibits pRb, together facilitating efficient immortalization. In contrast, Me3TSC is known to have been immortalized using SV40 large T antigen, which similarly promotes immortalization through functional inhibition of both p53 and Rb.

Although viral transduction enabled efficient immortalization of benign meningioma cell lines, concerns were inevitable regarding perturbation of the native genetic background. Such manipulations substantially alter cellular phenotypes and limit the interpretation of studies aimed at understanding the intrinsic biology of benign meningiomas. Furthermore, even Ben-Men-1, the most widely used benign meningioma cell line, was later found to harbor homozygous *CDKN2A/B* deletion, calling into question its suitability as a true benign model [67]. Thus, research on benign meningioma cell lines in this era highlights the inherent difficulty of establishing biologically faithful immortalized models.

#### 2010s: diversification of cell lines and refinement of culture conditions

The application of serum-free medium, the spontaneous immortalization of benign meningioma cells, and the establishment of malignant cell lines harboring *NF2* alterations characterize this era. Furthermore, genetically engineered meningioma mouse models have been established by the group of Kalamarides [40, 70], with subsequent derivation of tumor cell lines from these models, including the MGS1–3 series [70].

Surprisingly, most meningioma cell culture studies continued to rely on basal media such as MEM, DMEM, and RPMI 1640, all of which can be traced back to the 1950s [71]. Many of the essential requirements for meningioma cell growth remained poorly defined, prompting efforts to identify the minimal conditions necessary for sustained culture. This shift in perspective paved the way for the development of 3D culture systems and ultimately for the use of organoid models in the 2020s.

Neurobasal medium began to be applied by the Massachusetts General Hospital group in the late 2010s [72, 73]. They used Neurobasal medium supplemented with B27, N2, heparin, L-glutamine, EGF, FGF2, PDGF-BB, and antibiotics. Using this serum-free system, they successfully established tumorsphere cultures from malignant meningiomas. MN3 and MN8, both reported by this group, were derived from *NF2*-mutant anaplastic meningiomas.

KCI-MENG1 is notable as a benign meningioma cell line that underwent more than 70 passages without deliberate genetic manipulation [74]. It was established at Wayne State/Karmanos Cancer Institute, and although *NF2* was intact, the cells exhibited multiple translocations and high telomerase activity. NCH93, reported in 2019 by the University of Heidelberg, is another notable cell line characterized by an *NF2* frameshift deletion [75]. In contrast to the MGH group [72, 73], both groups establishing KCI-MENG1 and NCH93 used conventional DMEM-based culture conditions and developed monolayer 2D models with canonical *NF2* alterations, independent of artificial genetic immortalization [74, 75].

#### 2020s: expansion of 3D and organoid-based modeling

This era marked a new paradigm in the development of 3D culture systems and organoid platforms. In parallel, as multi-omic analyses became more widely established, they were increasingly incorporated into the evaluation of in vitro models [76–79].

Early in this period, a scaffold-free 3D spheroid model demonstrated that patient-derived meningioma cells could retain key histological, immunohistochemical, and mutational features of the parental tumors in three-dimensional culture [80]. Compared with matched two-dimensional cultures, the 3D spheroids showed enhanced epithelial-to-mesenchymal transition– and Notch signaling–related programs. In a separate study, a xeno-free hydrogel-based 3D model was established from fresh patient-derived meningioma cells [81]. Genome-wide DNA methylation analysis showed that this model largely preserved the methylation patterns and methylation-based classification of the parental tumors.

During this period, several groups successfully developed meningioma organoid models. In 2021, patient-derived meningioma organoids were generated from mechanically and enzymatically dissociated tumor tissues embedded in Matrigel, with preservation of somatic mutations, copy-number alterations, transcriptomic features, and DNA methylation patterns [82, 83]. Later, organoids established without enzymatic dissociation showed improved preservation of stromal and immune components, including endothelial cells and macrophages/microglia, while maintaining much of the parental genomic landscape [84]. Together, these studies showed that meningioma organoids can preserve not only tumor-intrinsic features but also part of the native microenvironment.

A further conceptual advance came in 2026, when a standardized tumor organoid model was applied to large-scale drug screening [85]. Rather than relying on conventional extracellular matrix-based organoid culture, this approach used controlled reaggregation of single-cell suspensions from fresh meningioma tissue to generate uniform organoids suitable for high-throughput testing. Using this system, Panobinostat, targeting HDAC1/2, was identified as a promising therapeutic agent, and a potential resistance mechanism involving the HDAC8–TGF-β–EMT axis was proposed.

At the same time, 3D models began to move beyond phenotypic recapitulation and toward functional discovery. In a spatial multi-omic study, 3D coculture models were integrated with CRISPRi and lineage tracing to explore therapeutic strategies addressing intratumor heterogeneity, emphasizing the need for systemic approaches that target multiple biological pathways [45].

Finally, this decade also brought progress in long-term culture systems, exemplified by the spontaneous immortalization of a benign meningioma under hypoxic conditions without genetic manipulation [86]. The resulting TKB-MEN2 line proliferated beyond the limits typically observed under normoxia, suggesting that hypoxia may attenuate senescence and thereby facilitate sustained expansion.

#### Classifications of meningioma cell lines: strengths and limitations

**Fig. 2:**
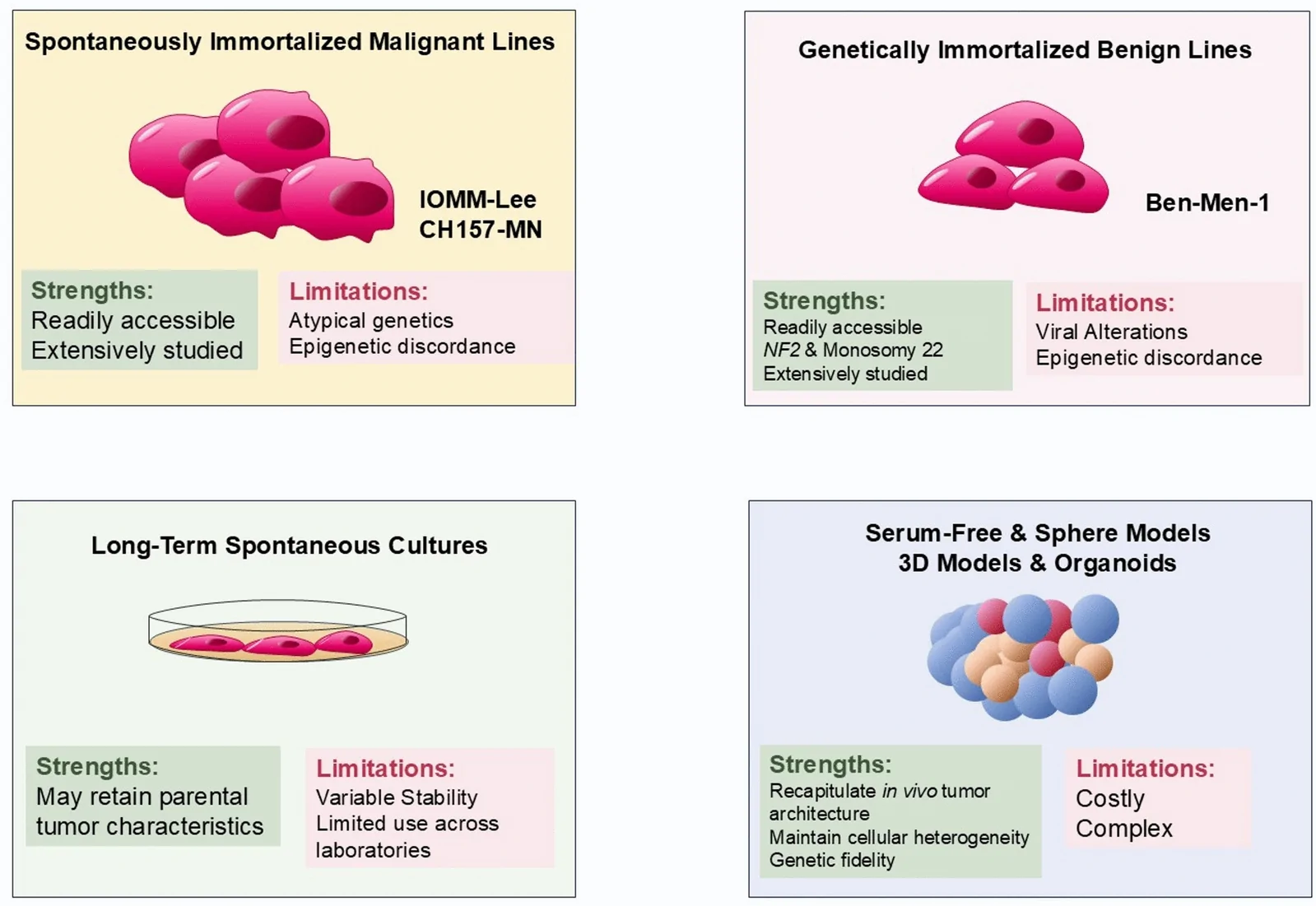
Strengths and limitations of in vitro meningioma models. While spontaneously immortalized malignant lines and genetically immortalized benign lines are widely used, they carry inherent limitations, including atypical genetic features and epigenetic discordance. Although long-term spontaneously immortalized cultures may retain parental tumor characteristics to some extent, they vary in stability and remain limited in use across laboratories. Three-dimensional models and organoids are gaining attention for their potential and fidelity to parental tissue, although they remain costly and technically complex

As reviewed above, meningioma models developed over the past four decades can be reclassified into five groups according to their method of establishment (Fig. 2):

(i) spontaneously immortalized malignant lines (e.g., IOMM-Lee and CH157-MN),
(ii) genetically immortalized benign lines (e.g., Ben-Men-1),
(iii) long-term cultures with spontaneous immortalization under specific conditions (e.g., TKB-MEN2),
(iv) serum-free or sphere-forming models, and
(v) 3D models and organoids.

Spontaneously immortalized malignant lines are widely used, readily accessible, and supported by a substantial body of literature. However, as discussed above, these lines often harbor atypical genetic backgrounds, and their epigenetic classifications are not always concordant with their malignant phenotypes. In the case of IOMM-Lee, its primary intraosseous origin further distinguishes it from the great majority of meningiomas. By contrast, Ben-Men-1 recapitulates the canonical combination of *NF2* alteration and monosomy 22, making it relatively faithful to the representative genetic background of benign meningioma. However, its homozygous deletion of *CDKN2A/B* would qualify it as WHO grade 3 under the current classification system, and hTERT-mediated immortalization itself introduces an additional layer of biological perturbation. More broadly, other virally immortalized lines also inevitably carry artificial genetic alterations. Thus, the meningioma cell lines most widely used to date all entail substantial limitations.

More recent models offer important advances. The establishment of TKB-MEN2 under hypoxic conditions is promising and the development of serum-free or sphere-forming cultures, as well as 3D models and organoids, has expanded the range of biologically informative systems. These newer models appear to preserve diverse molecular features more faithfully and have already enabled applications such as pathway-oriented analyses and large-scale drug screening. A comprehensive summary of currently available in vitro meningioma models and their molecular characteristics is provided in Table 1. Although issues remain regarding long-term propagation, standardization, and broad sharing across laboratories, these emerging platforms may help clarify aspects of meningioma biology that have been difficult to capture with conventional cell lines.

**Table 1:** Characteristics and molecular profiles of reported in vitro meningioma models

Author (year)	Country	Cell line	Grade	Genetic background	Patient	Anatomical site	Passage	Immortalization	Isolation
Tanaka (1989) [54]	Japan	KT21-MG1	NA (Malignant)	- 22, myc/fps amplification	47F	Falx	More than 2 years	Spontaneous	Tumor tissues were xenotransplanted into nude mice (KN-21), and removed from the subcutis later
Lee (1990) [48]	Taiwan	IOMM-Lee	NA (Malignant)	Intact 22, *NF*2; *CDKN2A/B* homozygous deletion, *TERT*p C228T, *BRAF* V600E; complex karyotype	61 M	Intraosseous	*P*>60	Spontaneous	Minced into tiny pieces approximately 1 mm in diameter
Tsai (1995) [56]	Taiwan	CH-157MN	NA	-22, *NF2* splice-site mutation (c.241-1G>T); 1q gain, 18 loss; *PTEN* truncating mutation, *TERT*p C228T, *NRAS* Q61K	41F (59F / 55F)	NA	*P*>40	Spontaneous	–
Pallini (2000) [60]	Italy	NA	WHO grade I, fibromatous	NA	49 M	Frontal convexity	NA	–	Fresh tissue was immediately placed in sterile phosphate-buffered saline. Under sterile conditions, the specimens were minced in 1 mm slices. After several washes with PBS in order to eliminate blood cells, the pieces were treated with 0.25% trypsin–EDTA solution at 37C for 20 min in 50 ml polypropylene centrifuge tubes. At the end of the incubation time, the partially digested tissue was mechanically dissociated with a pasteur pipette and centrifuged at 200xg for 10 min
		NA	WHO grade I, syncytial		52 M	Occipital convexity			
		NA	WHO grade II, atypical		33F	Falx			
		NA	WHO grade I, fibromatous		72F	Falx			
		NA	WHO grade I, syncytial		48 M	Parietal convexity			
		NA	WHO grade I, syncytial		68 M	Petrous bone			
		NA	WHO grade I, syncytial-angioblastic		61 M	Falx			
		NA	WHO grade I, angioblastic		58 M	Sphenoid wing			
		NA	WHO grade II, atypical		47 M	Sphenoid wing			
Akat (2003) [61]	Germany	HBL-52	WHO grade I, transitional	NA	NA	Optic canal	> 5 years	Spontaneous	–
Ishiwata (2004) [62]	Japan	HKBMM	NA (Malignant)	Model number of 48, 21p+, aneuploidy	-	Frontal lobe	>5 years	Spontaneous	Rinsed twice with the culture medium and minced with a sharp pair of scissors in the culture medium containing 600 Pronase Unit Dispase, and centrifuged at 900 rpm for10min
Puttmann (2005) [66]	Germany	Ben-Men-1	WHO grade I, meningothelial	- 22q	68F	Parietal falx	>23 months	Retroviral transduction of hTERT	At 20 min after resection, the tissue was cut into small pieces
Baia (2006) [69]	USA	SF4068	WHO grade I	NA	NA	NA	PD > 100	Grade I: hTERT + E6/7 Grade III: hTERT	Placed into DMEM + 10% FBS within 90 s following surgical resection, transported to the lab and dissected into 1 mm pieces Digestion with DNAse, Pronase and Collagenase for 30 min at 37 °C. The tissue fragment solution was filtered through a 100-micron filter, and the filtrate centrifuged at 1000 rpm for 10 min. Seeded at a density of 3 to 5 × 10^6^ cells per 100 mm dish
		SF4433	WHO grade I		NA	NA	PD >70		
		SF3061	WHO grade III		NA	NA	PD >200		
Cargioli (2007) [68]	USA	Me3TSC	WHO grade I, transitional	45, XX, t(1;5)(p36;q13), del (9)(p13), del(11)(p14); − 22	NA	NA	*P*≥28	Me3TSC: hTERT + SV40 large T antigen Me10T: hTERT	minced into 2- to 4-mm3 pieces and then plated into 75mm2 flasks with medium
		Me10T	WHO grade I, meningothelial	45, XX, − 22	NA	NA	*P*≥20		
Striedinger (2008) [91]	USA	MENII-1	WHO grade II	22q loss	NA	NA	NA	hTERT + E6/7	–
Michelhaugh (2015) [74]	USA	KCI-MENG1	WHO grade 1	*NF2* intact; Near triploid, multiple translocations including t(2;13)(q37;q22) and t(4;7)(q21;p13)	46F	Olfactory groove	*P* >70	–	Washed in PBS with 2 mM EDTA to remove blood and then chopped into fragments using a sterile single-edge razor blade. The fragments were washed in PBS without EDTA and digested with collagenase type IV
Nigim (2016) [72]	USA	MN3	NA (Malignant)	*NF2* (splice-site variant)	NA	NA	NA	Tumorsphere culture / Serum-free	Minced into small pieces and digested in 0.1% trypsin and DNAase for 40 min at 37 °C. After washes in Hank's Balanced Salt Solution, cells were cultured
Nigim (2019) [73]	USA	MN8	WHO grade III, anaplastic	*NF2*-mutant	NA	Intraventricular	NA	–	–
Jungwirth (2019) [75]	Germany	NCH93	WHO grade III, anaplastic	*NF2* (frameshift deletion; p.D130fs, p.D172fs, p.D213fs)	64 M	Parieto-occipital	NA	–	Mechanically dissected
Kim (2020) [76]	Korean	M1	WHO Grade I, meningothelial	NA	56F	Left sphenoid ridge	NA	–	Fresh Hank's Balanced Salt Solution at 4 °C. Tumor tissues were cut and trypsinized for 30 min with 0.1% trypsin at 37 °C. Cells were seeded at a density of 5 × 10^5^ cells /ml in a 6-well plate
		M2	WHO Grade I, meningothelial	NA	59F	Left posterior petrous	NA	–	
		M3	WHO Grade I, transitional	NA	62 M	Right frontal convexity	NA	–	
		M4	WHO Grade I, transitional	46, XX [19/30]; Tetraploid [11/30]	65F	Right frontotemporal convexity	NA	–	
		M5	WHO Grade II, atypical	46, XY [26\30]; 45, X, -Y [4/30]	57 M	Right parasagittal	NA	–	
Perepechaeva (2023) [77]	Russia	NA	WHO grade 1, meningothelial	NA	NA	NA	NA	－	Washed with PBS and crushed, and a 0.1% solution of type I collagenase was added and incubated for 39 min at 37 °C. Large pieces of tissue was removed and centrifuged at 1000 rpm for 5 min
Sun (2025) [79]	USA	LB3750	WHO grade 1	NA	NA	NA	*P*≤8	－	Minced, enzymatically dissociated with collagenase type I and dispase in 2 h, then dissociated via pipetting and plated in laminin-coated flasks
Trasaktaweesakul (2025) [78]	Thailand	SUT-MG9	WHO grade 1, meningothelial	NA	57F	Tuberculum sellae	*P*≤10	–	Rinsed in PBS to remove the DMSO and resuspended in a complete medium. The tissues were minced into 2-3mm3 pieces. Tissue fragments were transferred into a 15 mL tube. The fragments were resuspended in pre-warmed complete medium with collagenase solution at 1 mg/mL and incubated for 45 min at 37 °C with gentle stirring every 5 min. The digested tissues were sieved through 100 and 70 µm nylon filters. Cell suspensions were centrifuged and washed with complete medium
		SUT-MG12	WHO grade 1 with angiomatous and microcystic virant		70F	Frontal convexity	*P*≤10	–	
		SUT-MG14	WHO grade 1, meningothelial		63F	Falx	*P*≤10	–	
		SUT-MG16	WHO grade 1, meningothelial		64 M	frontal convexity	*P*≤10	–	
Ishikawa (2025) [86]	Japan	TKB-MEN2	WHO grade 1, transitional	*TERT*p-wild-type, *CDKN2A/B* intact	71 M	Falx	*P*>20	Establishment under hypoxia	Placed into ice-cold growth medium. The specimen was washed twice with Hank's balanced salt solution, and shredded in a 0.5 mL solution of 0.25% trypsin + 0.02% EDTA/PBS(-) using a razor scalpel, and incubated at 37°C for 30 min. 3 ml of GM was added, and dissociated by vigorous pipetting. After centrifugation (340xg for 5 min) at room temperature, the supernatant was removed, and GM was added. The cells were suspended and seeded onto a 60 mm dish

### Insights emerged from cell line studies

In silico studies based on clinical specimens have accumulated steadily and now provide a shared molecular framework for understanding meningioma biology. By contrast, wet-lab studies centered on functional assays have often focused on relatively specific targets, leaving much of the literature fragmented. Furthermore, as discussed in the previous section, classical cell line models do not fully recapitulate the biological complexity of patient tumors. Nevertheless, they have generated important mechanistic clues, with uneven levels of support from primary cultures, organoid models, and patient-level multi-omic studies.

With these considerations in mind, this section aims to synthesize insights derived primarily from cell line studies and place them in the context of recent large-scale multi-omic analyses.

#### Premise: mutational landscape and dysregulated pathways

Genetic alterations in meningioma are broadly categorized into *NF2*-mutant and non-*NF2* groups. Loss of the *NF2* tumor suppressor gene is the most prevalent alteration, occurring in approximately 60% of cases [4, 5, 87]. The *NF2* gene encodes merlin, a multifaceted scaffold protein that exerts inhibitory control over several cascades, including the Hippo, PI3K/Akt/mTOR, MAPK, and Wnt/β-catenin signaling. Merlin serves as a critical regulatory brake, and its deficiency drives tumorigenesis and tumor evolution.

In contrast, non-NF2 meningiomas are characterized by a diverse set of mutations, frequently referred to by the mnemonic TRAKLS (*TRAF7*, *AKT1*, *KLF4*, and *SMO*), alongside significant contributions from *PIK3CA* and *POLR2A* [4–7]. *AKT1* and *PIK3CA* converge onto PI3K/Akt/mTOR signaling, whereas *SMO* belongs to Hedgehog pathways.

Aggressive meningiomas are characterized by a distinct epigenomic landscape and by hypermetabolic and proliferative signatures [18, 19, 33]. Chromosome-scale genomic disruption is thought to be deeply intertwined with tumor evolution, although the contribution of each large-scale genomic rewiring event remains incompletely understood. Nevertheless, several key molecular phenomena have emerged, including the upregulation of FOXM1.

**Fig. 3:**
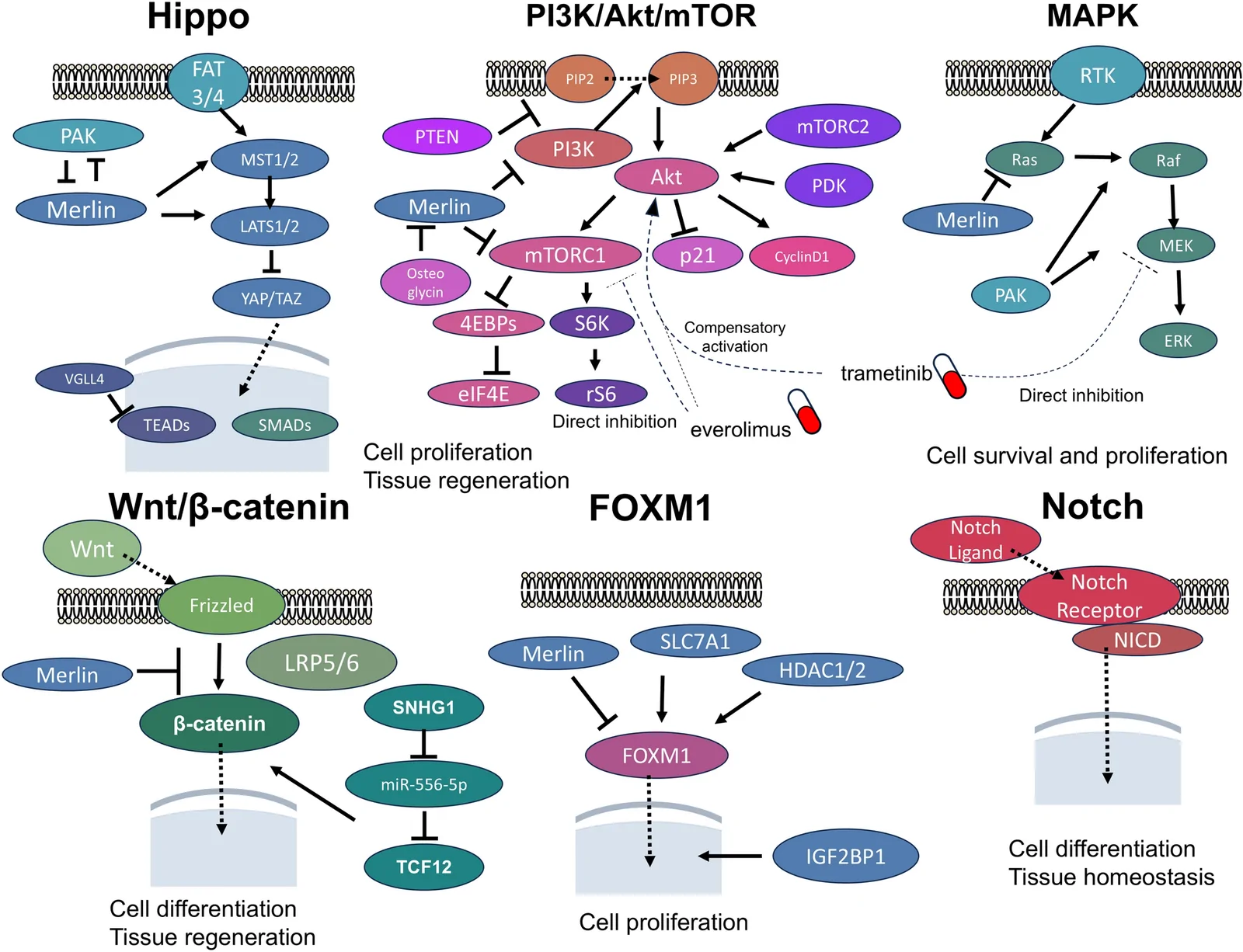
Key signaling networks in meningioma. Six major signaling networks are schematically illustrated: Hippo, PI3K/Akt/mTOR, MAPK, Wnt/β-catenin, FOXM1, and Notch. In each panel, canonical pathway components are combined signaling, with context-specific molecular interactions reported in meningioma in vitro studies, highlighting points of pathway activation, crosstalk, and dysregulation. Dashed arrows indicate pathway crosstalk and treatment-induced compensatory signaling, including AKT activation following MEK inhibition with trametinib. Collectively, these networks converge on core biological processes relevant to meningioma biology, including cell proliferation, survival, differentiation, tissue regeneration, and homeostasis

These observations support an integrated framework of meningioma signaling. At its core, merlin loss in *NF2*-mutant tumors functionally intersects with multiple major cascades, whereas non-*NF2* alterations more often converge on specific signaling nodes. In aggressive meningiomas, FOXM1 appears to represent a central downstream hub of proliferative rewiring, with Notch signaling emerging as an additional axis of interest. Hence, we organize prior functional studies, drawn largely from the cell line literature, into six major signaling pathways: (1) Hippo, (2) PI3K/Akt/mTOR, (3) MAPK, (4) Wnt/β-catenin, (5) FOXM1, and (6) Notch (Fig. 3).

#### Hippo

The Hippo signaling is a highly conserved signaling network that governs organ size and tissue regeneration [88]. This signaling pathway governs spindle positioning and the proper orientation of the cleavage plane during cell division [89]. Its activity is modulated by diverse upstream cues, including cell–cell adhesion, cell–matrix adhesion, and contractile tension generated by the actin cytoskeleton.

Merlin functions upstream of MST1/2 and LATS1/2 to restrain the activity of the downstream effectors YAP and TAZ. Upon release from this inhibitory constraint, YAP/TAZ translocates into the nucleus and cooperates with transcriptional regulators including TEAD and SMAD, thereby promoting the expansion of stem and progenitor cell populations. Although dysregulation of Hippo signaling has been described in many cancers, direct mutations affecting its core components are uncommon [90]. Meningioma is one of the few exceptions in which canonical Merlin dysfunction directly perturbs Hippo signaling.

Merlin-mediated growth control through YAP was first demonstrated in meningioma in 2008 [91]. Merlin was shown to mediate contact-dependent growth inhibition, and its loss led to nuclear accumulation of YAP, enhanced anchorage-independent growth, and increased S-phase entry; these effects were reversed by siRNA-mediated YAP inhibition.

Subsequent work further showed that cell density and extracellular matrix stiffness influence Merlin expression and YAP nuclear localization, consistent with a Merlin–YAP axis underlying contact inhibition of proliferation [92]. Accordingly, in *NF2*-intact meningiomas, tumor rims exhibit low Merlin expression with high nuclear YAP, whereas tumor cores show the opposite pattern.

Lead exposure has likewise been implicated in Hippo signaling dysregulation, accompanied by reduced Merlin expression, increased nuclear YAP localization, and enhanced tumor proliferation [93].

PAK, another component linked to this axis, promotes cell survival and suppresses apoptosis, and is reciprocally regulated with Merlin [94]. Merlin inhibits PAK activity, whereas PAK phosphorylates Merlin at Ser518 and attenuates its function. Hence, Merlin loss results in aberrant PAK activation. In meningioma cells, PAK inhibition has shown antitumor effects.

Further downstream, TEAD transcription factors have emerged as potential therapeutic targets in *NF2*-null meningioma [95]. Although YAP and TAZ are often discussed together, particularly in the schwannoma literature, they may play distinct roles. ALDH1A1 appears to be regulated by TAZ rather than by YAP.

More recently, however, a paradoxical pattern of YAP activity has been described [96]. Whereas benign *NF2*-mutant meningiomas conform to the canonical Hippo model, aggressive *NF2*-mutant tumors may instead exhibit reduced YAP activity. This apparent paradox has been attributed to concurrent genomic abnormalities that activate multiple signaling pathways, leading to upregulation of FAT3/4, which suppresses YAP, as well as VGLL4, which disrupts the YAP–TEAD interaction.

Beyond growth control, Hippo signaling may also be relevant to chromosome-scale genome evolution in meningioma through its role in spindle positioning and faithful cell division, although this connection remains insufficiently explored.

#### PI3K/Akt/mTOR

The PI3K/Akt/mTOR pathway is a major membrane-to-cytoplasm signaling axis involved in a broad range of cellular functions [97]. This pathway interacts with multiple growth factor receptors and downstream effectors, including 4EBPs, p21, cyclin D1, and S6K [98, 99]. Within this network, mTOR operates as the core catalytic subunit of two distinct complexes, mTORC1 and mTORC2 [100]. While mTORC1 primarily serves as a nutrient sensor that drives cell growth and metabolism, mTORC2 mainly controls cell survival, cytoskeletal organization, and the maximal activation of Akt. PTEN is a negative regulator of the PI3K/Akt/mTOR pathway, whereas merlin suppresses mTORC1 while promoting mTORC2 activity. In meningioma, this pathway can be activated directly by mutations in key components such as *AKT1* and *PIK3CA* [4–6], or indirectly by alterations in upstream negative regulators (merlin and PTEN) [11, 19].

Merlin loss was shown to directly activate mTORC1 independently of PI3K-Akt or MAPK signaling in meningioma [101]. Additional upstream inputs also converge on this pathway: HER2 stimulation promotes PI3K/Akt/mTOR-dependent proliferation and motility [102], osteoglycin may enhance meningioma progression through NF2 downregulation and AKT/mTOR activation [103], and hsa_circ_0004872 activates the pathway through the miR-190a-3p/PTEN axis [104]. In *AKT1* E17K-mutant meningiomas, pathway activation was associated with hexokinase 2 upregulation and reduced oxidative stress [105].

This signaling network is further shaped by feedback and pathway crosstalk. mTORC1-dependent negative feedback from S6K1 to AKT may limit therapeutic efficacy [101], whereas inhibition of the MAPK pathway with trametinib induces compensatory AKT activation, indicating reciprocal interaction between the PI3K/Akt/mTOR and MAPK pathways [55].

Accordingly, multiple therapeutic strategies have been explored, including inhibition of key pathway components [55], combination with somatostatin receptor blockade [98], AZD5363 for *AKT1* E17K-mutant tumors [106], third-generation mTOR inhibitors in NF2-SWN-associated meningioma [99], and brigatinib plus mTOR inhibition in *NF2*-deficient meningiomas [107].

Overall, these findings place the PI3K/Akt/mTOR pathway at the center of meningioma biology, while also indicating that its extensive feedback and pathway crosstalk may necessitate combinatorial rather than single-agent therapeutic strategies.

#### MAPK

MAPK, also referred to as ERK signaling, is a central mediator of cellular responses to mitogenic stimuli [108]. This kinase cascade is initiated by GTP-bound Ras at the plasma membrane, which activates Raf, followed by sequential phosphorylation of MEK and ERK. MAPK (ERK) regulates G1/S cell-cycle progression and suppresses apoptosis. Its dysregulation contributes to tumor growth, invasion, and metastatic progression.

MAPK signaling emerged as a relevant pathway in meningioma as early as the 2000s [109]. Subsequent studies indicated that its role should not be viewed in isolation but rather within a complex signaling architecture involving extensive crosstalk with the PI3K/Akt/mTOR and Notch pathways [110]. This conceptual shift has supported growing interest in multitarget kinase inhibition as a therapeutic strategy for meningioma [111, 112]. Functional studies demonstrated that Ras inhibition substantially reduced MAPK and AKT phosphorylation and suppressed proliferation in human meningioma cells, highlighting the close coupling of these pathways [113].

Merlin was shown to interact with Ras and RasGAP, and this regulatory linkage was implicated in growth suppression [114]. Within the non-*NF2* context, *TRAF7* loss activated MAPK signaling and induced *KLF4*-dependent transcriptional programs that normally restrain small GTPase activity [36]. Furthermore, coexisting *KLF4* mutations disrupted this feedback restraint and cooperatively promoted tumorigenesis.

Of note, recent multi-omic study incorporating spatial transcriptomics with unprecedented resolution has refined the view regarding this cascade [45]. Although both MAPK and PI3K/Akt/mTOR signaling remain central to meningioma biology, recurrent tumors appear to exhibit a shift toward predominant PI3K/Akt/mTOR activation with relative suppression of MAPK signaling. The relationship between the two pathways may be reconfigured during tumor recurrence and progression.

#### Wnt/β-catenin

Wnt/β-catenin signaling is involved in cellular differentiation and adult tissue regeneration [115]. β-Catenin is constitutively degraded in the absence of Wnt stimulation. Upon Wnt ligand binding to Frizzled, the co-receptors LRP5/6 are recruited, leading to stabilization and nuclear translocation of β-catenin, which then forms a transcriptional complex to regulate target gene expression. Representative downstream target genes include *MYC*, *CCND1*, *AXIN2*, and *CD44* [115–117]. While direct mutations in components of this pathway are well recognized in colorectal and hepatocellular cancers, meningioma-associated mutations do not usually affect these components directly, but rather activate the pathway indirectly [115].

Although the relevance of Wnt/β-catenin signaling to meningioma emerged later than in many other tumor types, an early exploratory study reported that several natural compounds suppressed meningioma cell growth together with reduced expression of Wnt/β-catenin-related components, providing preliminary support for pathway involvement [118]. Subsequent studies addressed the pathway more directly. In *NF2*-mutant settings, inhibition of CBP/β-catenin-associated transcription suggested that Wnt/β-catenin-related transcriptional activity may represent a therapeutic vulnerability in merlin-deficient meningiomas [119]. In contrast, in *NF2*-intact tumors, Merlin was proposed to regulate Wnt/β-catenin signaling in a phosphorylation-dependent manner, with serine 13 dephosphorylation enhancing Wnt/β-catenin activity and the phosphorylated state being associated with favorable clinical behavior [43]. Further layers of regulation have been suggested, including association with a Meflin-high state [120] and an SNHG1/miR-556-5p/TCF12 feedback loop [121].

Overall, current evidence supports a role for Wnt/β-catenin signaling in meningioma, but its mechanistic directionality and clinical significance remain incompletely defined.

#### FOXM1

FOXM1 is a pro-mitotic transcription factor and a central regulator of proliferative programs in aggressive meningiomas [16]. Its target gene network, particularly cell-cycle-related genes, is enriched in high-grade tumors and increased FOXM1 expression correlates with poor clinical outcomes.

Functional studies using meningioma cell lines and primary cultures have supported a direct role for FOXM1 in malignant phenotypes. Genetic or pharmacologic inhibition of FOXM1 suppressed proliferation [16], whereas subsequent knockdown and overexpression experiments further linked FOXM1 to proliferation, invasion, migration, angiogenesis, and cell cycle progression [17]. Mechanistically, these effects were connected in part to β-catenin signaling and loss of merlin was also reported to promote FOXM1 stabilization together with enhancement of Wnt/β-catenin activity [119].

Recent work has further placed FOXM1 within a broader regulatory network. IGF2BP1 has been reported to stabilize FOXM1 target transcripts in hypermitotic meningiomas [122], whereas dual repression of HDAC1 and HDAC2 efficiently suppresses FOXM1 and reduces the viability of high-grade meningioma cell lines [123]. SLC7A1 has also been proposed as an additional upstream regulator [124].

Together, these studies support a model in which FOXM1 functions as a central transcriptional hub linking proliferative output to multiple upstream oncogenic inputs.

#### Notch

Notch signaling is a juxtacrine cell–cell communication pathway involved in cell differentiation and tissue homeostasis [125]. Membrane-bound Notch ligands interact with the transmembrane Notch receptor on adjacent cells. This leads to cleavage of the receptor and release of the Notch intracellular domain (NICD). NICD then translocates to the nucleus, where it activates the transcription of target genes.

The involvement of Notch signaling in meningioma was first reported by the University of California, San Francisco group in 2005 [126]. They showed dysregulation of this pathway in meningioma and demonstrated that inhibition of the γ-secretase complex reduced cell survival. The same group subsequently reported that activation of Notch signaling through overexpression of HES1 and NICD induced tetraploidy and chromosomal instability [127]. More recently, the group further highlighted the importance of NOTCH3-positive perivascular stem-like cells in meningioma biology [42]. The blockade of the extracellular region of NOTCH3 with a specific antibody suppressed tumor growth, whereas NICD overexpression promoted cell proliferation, angiogenesis, and radioresistance. In 2025, NOTCH3 signaling was further implicated in metabolic reprogramming and ferroptosis in aggressive meningiomas [128].

These observations suggest a possible link between Notch dysregulation and chromosome-scale genomic evolution in meningioma. In particular, the association of Notch activation with tetraploidy and chromosomal instability makes this pathway of interest in future studies of aneuploidy and large-scale karyotypic remodeling.

### Future direction: lessons from other cancer models

Meningioma is characterized by highly recurrent CNAs, and its progression appears to parallel chromosome-scale genome evolution [26]. As discussed above, Hippo signaling regulates proper spindle assembly and cleavage-plane orientation during cell division [89], whereas Notch signaling has been implicated in tetraploidization and chromosomal instability [127]. While the Hippo and Notch signalings hold particular promise for elucidating the mechanisms underlying chromosome-scale genomic evolution in meningioma, studies directly connecting these regulatory pathways to its dynamic genomic evolution remain scarce.

In cancer biology, aneuploidy has long been recognized as a hallmark of cancer since Boveri’s seminal observations in the early twentieth century [129]. Since then, numerous mechanistic studies, many of them centered on cell line models, have investigated how aneuploidy arises and reshapes tumor evolution [130–133]. More recently, whole-genome doubling (WGD) has emerged as a major driver of karyotypic evolution [134]. Experimental systems based on p53/Rb deficiency, mitotic failure, or telomere crisis have enabled direct modeling of genome doubling and have provided mechanistic insight into how telomere dysfunction promotes cancer genome evolution [135]. Long-term propagation of tetraploid cells derived from diploid colon cancer cell lines has further illustrated how WGD fuels genomic diversification [136], and recent work has suggested that WGD can also induce large-scale rewiring of 3D chromatin architecture in p53-deficient cells [137]. However, such experimental paradigms have rarely been applied to meningioma, despite the growing recognition that chromosome-scale genomic evolution is central to its progression.

Furthermore, the recurrent chromosome-scale disturbances that define the canonical CNA patterns of meningioma remain poorly understood at the mechanistic level. Beyond mitotic segregation errors, replication stress is increasingly recognized as a major source of chromosomal aberrations, arising from DNA double-strand breaks and transcription–replication conflicts [131, 138]. For instance, Cyclin E induction has been shown to provoke transcription–replication conflicts and DNA damage in cell models [139], while human cell-based plasmid systems have clarified regulatory mechanisms governing S-phase R-loops and how perturbed replication can lead to widespread genomic instability [140].

These DNA damage response mechanisms are also directly relevant to therapeutic response. Radiotherapy remains an established nonsurgical treatment modality for meningioma, yet the molecular determinants of radiosensitivity and resistance remain incompletely understood. In malignant meningioma models, irradiation induces checkpoint activation and G2/M cell-cycle arrest, which may facilitate DNA repair and subsequent cellular recovery [141]. Key regulators of DNA damage sensing and repair, including ATM, ATR, PARP, RAD51, and BRCA1/2, may therefore influence radiotherapy response [142, 143]. Although direct evidence in meningioma remains limited, PARP1 expression [144], and modulation of RAD51 and DNA-PKcs activity [145], have been associated with tumor grade or experimental radiosensitization. MicroRNAs may provide an additional regulatory layer, as distinct microRNA profiles [146] and miR-221/222 signaling [147] have been linked to radiosensitivity in meningioma models. Further studies using molecularly characterized cell lines, primary cultures, and organoid systems will be required to define these pathways and identify rational treating strategies.

Together, these mechanistic approaches may open new avenues for understanding chromosome-scale genome evolution and therapeutic response in meningioma.

## Conclusion

The biology of meningioma has been substantially clarified by omics-based studies, while mechanistic studies have offered important insights into specific pathways and biological processes. Both approaches are indispensable: omics-based analyses define associations at scale, whereas functional studies are required to establish direct causality. Bridging these two perspectives will be essential for uncovering the missing mechanistic links in meningioma biology and for translating molecular insights into effective therapeutic strategies.

## Data Availability

Not applicable.

## Abbreviations

3D: Three-dimensional
CNA: Copy-number alteration
NICD: Notch intracellular domain
WGD: Whole-genome doubling
WHO: World Health Organization
